# Identifying Attitudes Toward and Acceptance of Osteopathic Graduates in Surgical Residency Programs in the Era of Single Accreditation: Results of the American College of Osteopathic Surgeons Medical Student Section Questionnaire of Program Directors

**DOI:** 10.7759/cureus.22870

**Published:** 2022-03-05

**Authors:** Matthew A Heard, Sara E Buckley, Bracken Burns, Kristen Conrad-Schnetz

**Affiliations:** 1 General Surgery, East Tennessee State University, Johnson City, USA; 2 School of Osteopathic Medicine, University of the Incarnate Word School of Osteopathic Medicine, San Antonio, USA; 3 Trauma/Critical Care, East Tennessee State University, Johnson City, USA; 4 Surgery, South Pointe Hospital Cleveland Clinic Foundation, Cleveland, USA

**Keywords:** thoracic surgery, plastic surgery, urology, vascular surgery, otolaryngology, orthopedic surgery, neurological surgery, general surgery, resident selection, surgical residency

## Abstract

Purpose

The purpose of this study was to quantify the number of surgical programs currently training osteopathic residents and to solicit advice for current osteopathic medical students who are interested in pursuing a surgical residency.

Methods

A questionnaire was sent to all listed Electronic Residency Application Service® (ERAS®) email contacts for the following specialties: General Surgery, Neurological Surgery, Orthopedic Surgery, Otolaryngology, Urology, Integrated Vascular Surgery, Integrated Plastic Surgery, and Integrated Thoracic Surgery. The questionnaire was sent a total of three times.

Results

Two hundred sixty-four of the 1,040 surgical residency programs responded to the questionnaire. Of these responses, 19% were formerly American Osteopathic Association (AOA) accredited programs. About 47.3% of responding programs indicated they are not currently training an osteopathic physician. One hundred thirteen programs provided additional comments on how osteopathic medical students may improve the competitiveness of their residency applications. These comments included increasing volumes of research activities, performing well on the United States Medical Licensing Exam (USMLE), and completing a sub-internship in the desired field or at a specific institution.

Conclusion

Osteopathic students still face many barriers to matching into surgical residencies. This study provides concrete steps students may take to increase the competitiveness of their application.

## Introduction

As osteopathic medical schools have expanded, osteopathic medical students now make up greater than twenty-five percent of all matriculating medical students, naturally leading to an increase in osteopathic applicants to competitive residency programs [1]. Despite the increase in applicant numbers, osteopathic medical students do not match into surgical specialties at the same rate as their allopathic counterparts, most recently evidenced in the 2021 National Residency Match Program (NRMP) match results [2,3]. Additionally, Beckman and Speicher noted in a 2020 article that surgical specialties are the least likely to train osteopathic physicians [4].

It is important to note that the year 2020 marked the first year where all applicants to post-graduate medical and surgical training participated in a single match process. Prior to single accreditation, osteopathic graduates had the option of participating in either the NRMP or the American Osteopathic Association (AOA) match. It is unclear what the effects will be on osteopathic physicians applying for and matching into surgical specialties with single accreditation now being completed.

The primary goal of this study was to characterize attitudes of surgical residency program decision makers toward osteopathic applicants by assessing the prevalence of osteopathic residents in their programs. A secondary goal was to provide recommendations for osteopathic applicants to increase the competitiveness of their eventual applications to surgical residency programs.

## Materials and methods

This study was determined to be an IRB exempt study by the institutional review board at East Tennessee State University. In October 2020, as a part of the American College of Osteopathic Surgeons (ACOS) Strategic Planning efforts, the ACOS Medical Student Section sent a questionnaire to the listed email contact for each surgical residency program in the Electronic Residency Application Service® (ERAS®). Responses were requested via email from representatives of residency programs in General Surgery, Neurological Surgery, Orthopedic Surgery, Otolaryngology, Urology, Integrated Vascular Surgery, Integrated Plastic Surgery, and Integrated Thoracic Surgery. Emails were sent to the listed contacts separated out into respective specialty groups using the blind carbon copy feature. Three separate attempts were made by email each month from October to December 2020 to obtain the data. Responses were stored in Google Forms.

The questionnaire requested answers to the following items: institution name, specialty, location, respondent name, and role. Participants were asked for responses to the following: (1) Do you have any osteopathic residents in your program? If yes, how many? (2) Do you find graduation from an osteopathic college of medicine a strength? (3) If the answer to either of the above questions is “no,” what can osteopathic medical students do to be more competitive for your program?

Analysis was performed to determine if the program is currently training an osteopathic physician, and the percentage of responding programs without a current osteopathic resident was calculated. Microsoft Excel (Microsoft Corp., Redmond, Washington, USA) was then used to generate a geographic heat map of the number of programs currently training osteopathic physicians by state. Additional comments given by the respondents were then assessed in order to appropriately categorize the responses.

## Results

Overall results 

A response was received from 264 (25%) of the 1,040 surgical residency programs participating in the 2021 NRMP cycle across the aforementioned specialties. Program directors made up 28.3% of the responses, with the remaining responses being by program coordinators. Roughly 19% of responses (50 of 264) were from programs formerly accredited by the AOA. Overall, 47.3% of responses indicated their program is not currently training an osteopathic physician. Table 1 provides a summary of the results from each surgical specialty.

**Table 1:** Results summary of responses for each surgical specialty. D.O.: Doctor of Osteopathic Medicine; AOA: American Osteopathic Association.

Specialty	Overall programs responding (%)	Programs without a D.O. (%)	Former AOA responses (%)
All	25.4	47.3	19.3
General surgery	23.3	15.1	27.4
Orthopedic surgery	24.6	37.8	33.3
Neurological surgery	28.7	66.7	9.1
Otolaryngology	28.8	64.7	20.6
Urology	29.7	56.1	14.6
Plastic surgery (integrated)	21.3	82.4	0.0
Thoracic surgery (integrated)	9.1	66.7	0.0
Vascular surgery (integrated)	26.7	62.5	0.0

**Figure 1:**
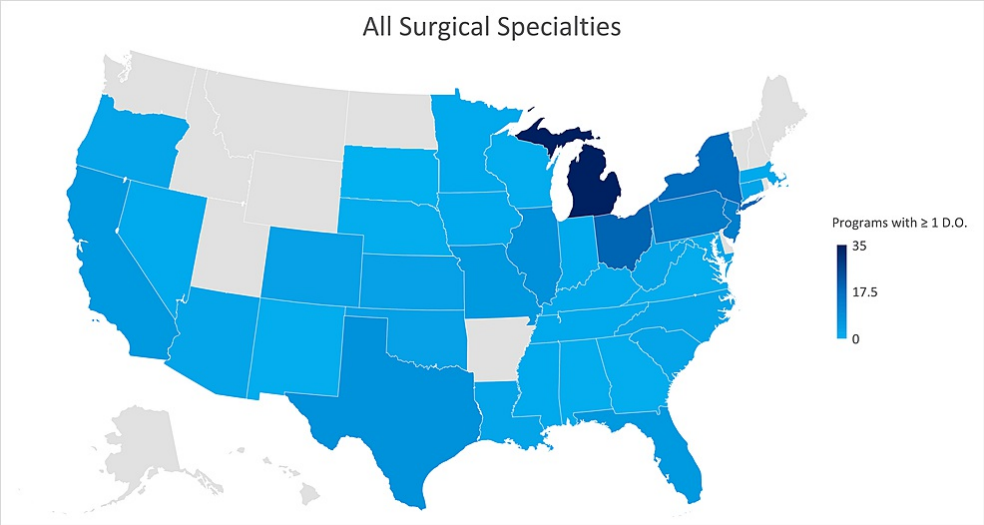
Geographic heat map showing the relative prevalence of all surgical residency programs in each state indicating osteopathic physicians currently training in their program. The map was created in Microsoft Excel (Redmond, Washington).

Figure 1 shows a heat map graphically indicating the states of Michigan, New York, and Ohio as those having a larger number of programs currently training osteopathic physicians.

**Figure 2:**
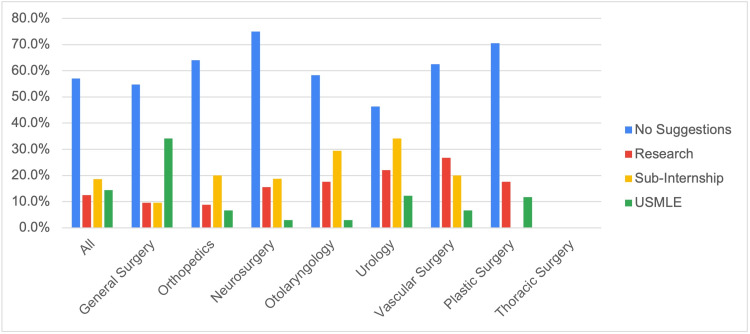
Reported suggestions for osteopathic medical students and graduates to increase their competitiveness in their desired specialty.

Of all the responses to the questionnaire, 38 programs (12.5%) suggested students should perform more research. Forty-five programs (18.9%) place emphasis on osteopathic students completing a sub-internship in their desired field or program to increase their competitiveness. Finally, 38 programs (14.4%) recommended that students complete and/or perform well on the United States Medical Licensing Exam (USMLE) in addition to the Comprehensive Osteopathic Medical Licensing Exam (COMLEX). Suggestions regarding how osteopathic students can become more competitive were not provided by 143 programs (54.1%). Figure 2 provides a graphical representation of the recommendations from respondents for each surgical specialty. Table 2 provides the percentage of responses provided by residency programs formerly accredited by the American Osteopathic Association.

**Table 2:** Suggestions received from surgical residency programs formerly accredited by the American Osteopathic Association as a proportion of all responses. AOA: American Osteopathic Association; USMLE: United States Medical Licensing Examination.

	Responses from former AOA programs as proportion of all responses		
Keyword	Research	Sub-Internship	USMLE
Specialty			
All	5/38 (13.5%)	10/45 (22.2%)	2/38 (5.2%)
General surgery	2/7 (28.5%)	2/7 (28.5%)	2/25 (8%)
Orthopedic surgery	0/4 (0%)	4/9 (44%)	0/3 (0%)
Neurological surgery	1/5 (20%)	1/6 (16%)	0/1 (0%)
Otolaryngology	0/6 (0%)	1/10 (10%)	0/1 (0%)
Urology	2/9 (22%)	3/14 (21.4%)	0/5 (0%)
Vascular surgery (Integrated)	0/4 (0%)	0/3 (0%)	0/1 (0%)
Plastic surgery (Integrated)	0/3 (0%)	0/0 (0%)	0/2 (0%)
Thoracic surgery (Integrated)	0/0 (0%)	0/0 (0%)	0/0 (0%)

General surgery

**Figure 3:**
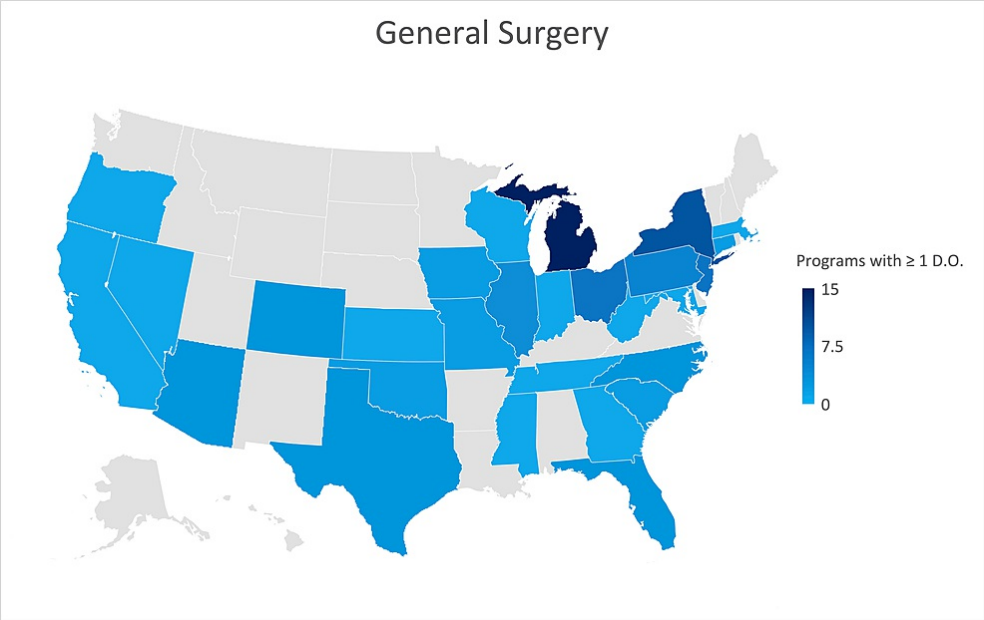
Geographic heat map showing the relative prevalence of general surgery residency programs in each state indicating osteopathic physicians currently training in their program. The map was created in Microsoft Excel (Redmond, Washington).

A response was received from seventy-three of the 313 general surgery residency programs participating in the 2021 NRMP cycle, or approximately 23.3% of all programs in the United States. Roughly 27% of responses were from programs formerly accredited by the AOA. Eleven responses (15%) indicated that their program was not currently training an osteopathic physician. Michigan, New York, and Ohio are the states that currently have a larger number of programs training osteopathic physicians (Figure 3).

Orthopedic surgery

**Figure 4:**
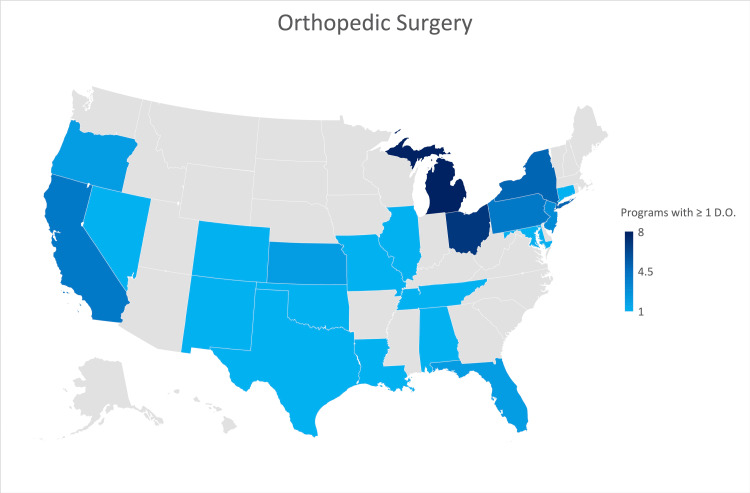
Geographic heat map showing the relative prevalence of orthopedic surgery residency programs in each state indicating osteopathic physicians currently training in their program. The map was created in Microsoft Excel (Redmond, Washington).

A response was received from forty-five of the 183 orthopedic surgery residency programs participating in the NRMP (24.6% of all programs). Thirty-three percent of responses were from programs formerly accredited by the AOA. Seventeen (37.8%) of the responses indicated that their program was not currently training an osteopathic physician. A heat map (Figure 4) graphically indicates that the states of Michigan, New York, Ohio, Pennsylvania, and California are those that have a larger number of programs currently training osteopathic physicians.

Neurological surgery

**Figure 5:**
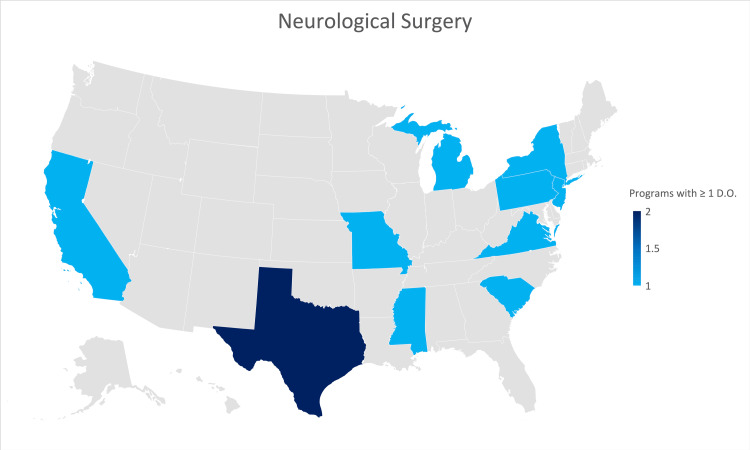
Geographic heat map showing the relative prevalence of neurological surgery residency programs in each state indicating osteopathic physicians currently training in their program. The map was created in Microsoft Excel (Redmond, Washington).

A response was received from thirty-three of the 115 Neurological Surgery residency programs (28.7% of all programs) participating in the 2021 NRMP. Three of thirty-three responses (9%) were from programs formerly accredited by the AOA. Twenty-two (66.7%) of the responses indicated that their program was not currently training an osteopathic physician. The state identified as training the largest number of osteopathic physicians was Texas (Figure 5).

Otolaryngology

**Figure 6:**
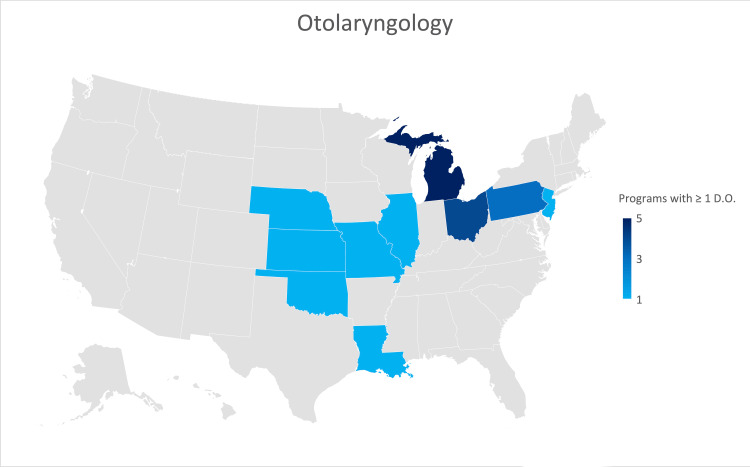
Geographic heat map showing the relative prevalence of otolaryngology residency programs in each state indicating osteopathic physicians currently training in their program. The map was created in Microsoft Excel (Redmond, Washington).

A response was received from thirty-four of the 118 (28.8%) Otolaryngology residency programs. Seven (20.6%) of thirty-four responses were from programs formerly accredited by AOA. Twenty-two (64.7%) of the responses indicated that their program was not currently training an osteopathic physician. Michigan, Ohio, and Pennsylvania have the largest number of programs currently training osteopathic physicians (Figure 6).

Urology

**Figure 7:**
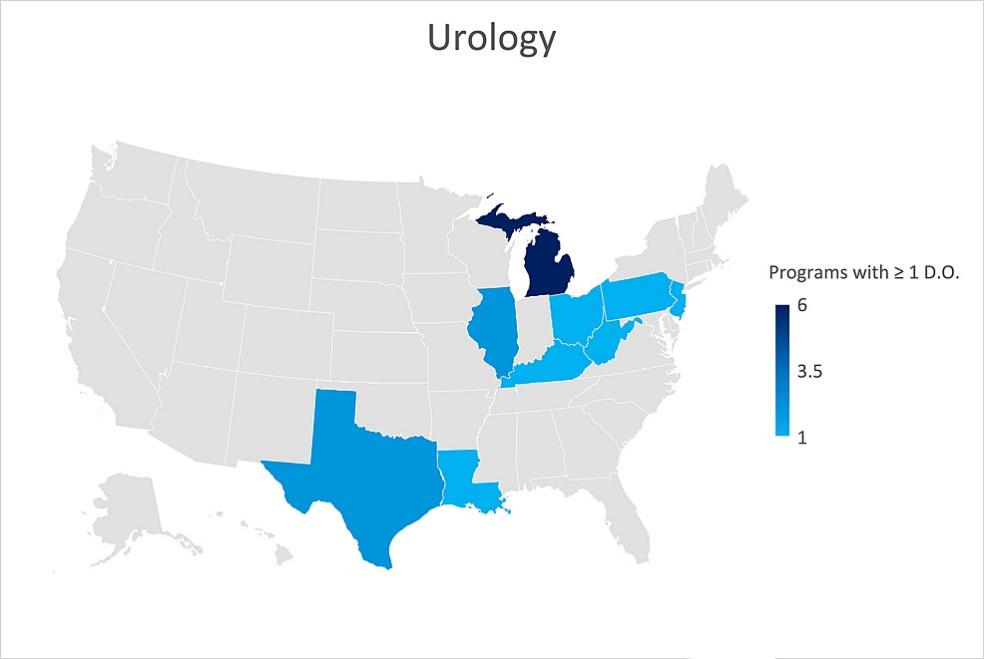
Geographic heat map showing the relative prevalence of urology residency programs in each state indicating osteopathic physicians currently training in their program. The map was created in Microsoft Excel (Redmond, Washington).

A response was received from forty-one (29.7%) of the 138 Urology residency programs. Six (14.6%) were from programs formerly accredited by the AOA. Twenty-three (56.1%) of the responses indicated their program was not currently training an osteopathic physician. The states identified as training the most osteopathic physicians were Michigan, Illinois, and Texas (Figure 7).

Vascular surgery (integrated)

**Figure 8:**
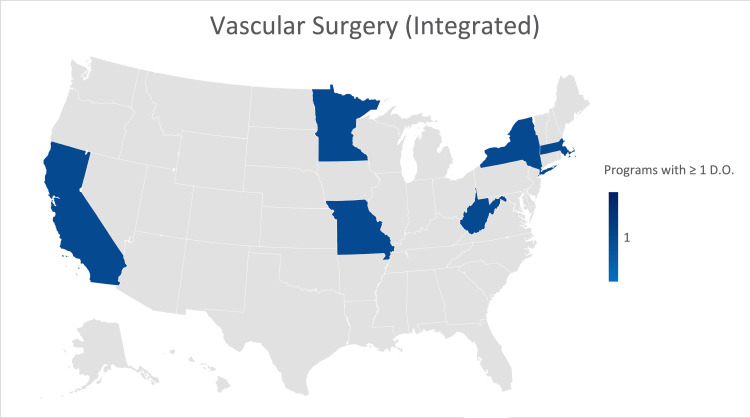
Geographic heat map showing the relative prevalence of integrated vascular surgery residency programs in each state indicating osteopathic physicians currently training in their program. The map was created in Microsoft Excel (Redmond, Washington).

A response was received from 16 (26.6%) of the 60 Integrated Vascular Surgery residency programs. No programs were formerly accredited by the AOA. About 62.5% indicated that their program was not currently training an osteopathic physician. Analysis showed the states of California, Minnesota, Missouri, West Virginia, New York, and Massachusetts each have a single program that is currently training an osteopathic physician (Figure 8).

Plastic surgery (integrated)

**Figure 9:**
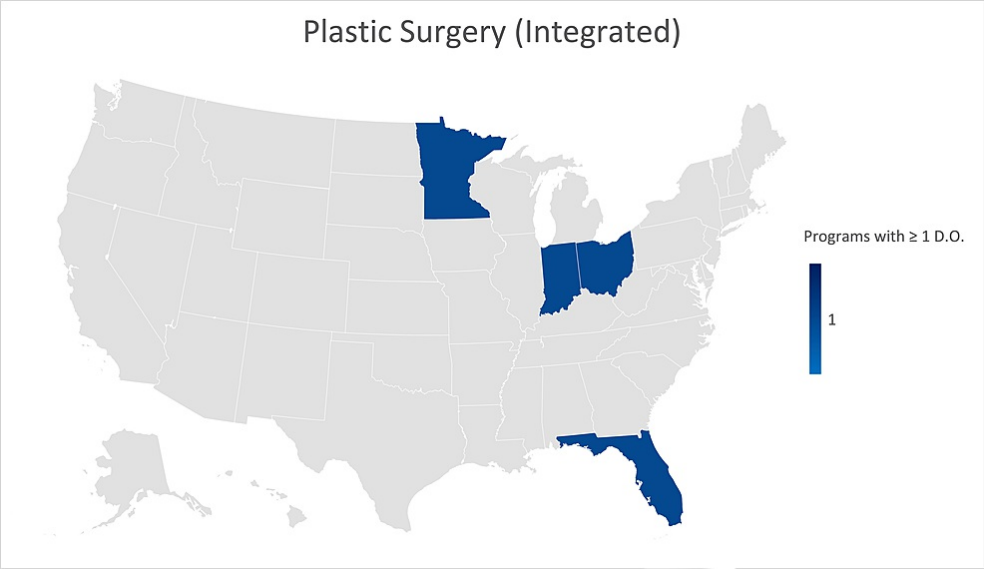
Geographic heat map showing the relative prevalence of integrated plastic surgery residency programs in each state indicating osteopathic physicians currently training in their program. The map was created in Microsoft Excel (Redmond, Washington).

Seventeen (21.25%) of the 80 Integrated Plastic Surgery residency programs provided responses. No programs were formerly accredited by the AOA. Fourteen (82.3%) of the responses indicated that their program was not currently training osteopathic physicians. Minnesota, Indiana, Ohio, and Florida each have a single program that is currently training an osteopathic physician (Figure 9).

Thoracic surgery (integrated)

Three (9%) of the 33 Integrated Thoracic Surgery residency programs responded. No programs were formerly accredited by the AOA. Two of the responses indicated that their program was not currently training an osteopathic physician. A heat map was not generated as only one program had given an affirmative response. Kentucky was the only state identified from respondents as having an osteopathic resident trainee.

## Discussion

The goal of the current study was to provide specialty-specific advice to medical students directly from residency programs in regards to the residency application process. Both the literature and the NRMP outcomes data align closely with the responses to our questionnaire, as both matched osteopathic and allopathic medical students shared similar USMLE step 1 and 2 scores and volume of research experience [5,6].

Schlitzkus et al. (2012) highlight their study comparing the characteristics of allopathic and osteopathic applicants to an allopathic general surgery residency program. Interestingly, their results showed no significant differences between allopathic and osteopathic applicants, even noting that osteopathic applicants averaged a significantly higher USMLE step 1 score compared to their allopathic counterparts [7]. These results suggest that other residency programs may have similar demographics in their applicants that would only be discovered with in-depth analysis of those who apply. However, despite these similarities, osteopathic applicants continue to have difficulties matching to certain residency programs and specialties [3]. Additionally, Etheart et al. discovered that 27 of the 48 general surgery residency programs previously accredited by the AOA were under the direction of MD program directors [3]. It is unclear if this finding has an impact on osteopathic applicants' matching into surgical residency programs, but it should be considered as a potential factor. The disparity in match rates despite similar applicant characteristics may suggest a continued bias against osteopathic medical students applying to competitive surgical specialties that should be addressed at the individual program level.

In a 2008 study, Melendez et al. determined that the most important factors for resident selection were the interview, USMLE step I scores, letters of recommendation, and alpha omega alpha (AΩA) membership [8]. Additionally, participation in basic science and clinical research was considered an important secondary factor. Naples et al., in a 2020 article, determined that attitudes toward completion of an “away” audition rotation or sub-internship at the student’s home institution were commonly recommended and viewed similarly at both academic and community-based general surgery residency programs [9]. The results of these studies align closely with the results of the current study. The similar results validate the suggestions seen above, suggesting that osteopathic medical students should take and/or perform competitively on the USMLE, perform and publish research, and complete sub-internship rotations at the residency programs they are interested in.

Likewise, fourth-year medical students also view the sub-internship, also known as an audition rotation, as an important piece of their residency application. According to Winterton et al., as many as 58.7% of fourth-year medical students completed an audition rotation in their 2016 study [10]. Jacobson et al. also showed that the applicants who completed these rotations had a significantly higher match rate compared to those who did not [11]. In the midst of the COVID-19 pandemic, the American Association of Medical Colleges (AAMC), recommended that students should complete rotations within their own institutions as much as possible [12]. This created a large disparity as traditionally osteopathic medical schools are not necessarily affiliated with teaching hospitals that have graduate medical education programs. Of note, 75% of osteopathic medical schools are without an affiliated General Surgery Residency program [13]. Students without a home program may be limited in their ability to complete audition rotations. These limitations may be financial, family-based, or in light of the COVID-19 pandemic, limited due to public health measures. Barriers to formal rotations at Accreditation Council for Graduate Medical Education (ACGME) residency programs may be alleviated if medical schools were to sponsor their own residency programs and/or form formal agreements with programs in their region to assure not only educational equity for students, but also post-graduate training opportunities.

As suggested by this study and multiple surgical specialty organizations, research is an important selection factor for residency programs. Upon review of the US News and World Report research rankings, osteopathic medical schools do not appear to have adequate research opportunities when compared to larger institutions, suggesting that osteopathic applicants have to look elsewhere for this experience [14]. Though these rankings do not address the quality of research performed, research experiences foster connections and mentorship that can have a significant impact on the experience of a residency applicant. Given an emphasis on research at competitive residency programs, studies suggest that external research fellowships are becoming increasingly utilized for applicants to increase their competitiveness. In plastic surgery, it was reported that those who completed such a fellowship were more likely to match into an integrated plastic surgery residency, while a study comparing results to orthopedic surgery programs did not find such an association [15,16]. Additionally, in a 2019 study, Matthews et al. discovered a significant difference in research metrics between matched and unmatched applicants, as well as osteopathic and allopathic applicants. When specialty-specific differences were explored for General Surgery and Orthopedic Surgery, there were significant differences in research experiences in those allopathic applicants who were matched compared to those who went unmatched into a residency program. No such difference was discovered for osteopathic applicants, indicating that applicants may have solely applied to programs that place less emphasis on research [17]. These results suggest that osteopathic medical schools should have a greater emphasis on research opportunities to provide their students with the greatest opportunity to succeed.

There are many components to a residency application packet, however, and the required board exams remain a significant factor in the decision to interview and rank applicants [18]. Allopathic medical students are required to take the USMLE, whereas osteopathic medical students are required to take the COMLEX. While the American Medical Association House of Delegates has declared equality between the two exams, USMLE scores are a common requirement, in particular for surgical residency programs [19]. Not surprisingly, one of the largest discussion points among osteopathic medical students regards the decision to take the USMLE exam in addition to the COMLEX exams in an effort to fulfill these application requirements. There are multiple studies in the literature that have attempted to calculate a score convertor for both USMLE step 1 and step 2 clinical knowledge (step 2CK) to allow direct comparison of those without USMLE scores to those who do. These studies have been found to under predict USMLE scores and have not been validated in large population studies [20-22]. Additionally, the examinations themselves have differing goals and content, essentially forcing osteopathic medical students interested in competitive specialties to take the USMLE [23]. The increased registration costs as well as stress associated with sitting for multiple high-stakes examinations place unnecessary expectations on osteopathic medical students alone [23].

With the USMLE step 1 exam transitioning to pass/fail scoring in the near future, there are concerns that this change will have disproportionate impacts on osteopathic medical students and international medical graduates who aim for high step 1 scores to become competitive applicants [24]. This change is likely to lead to further emphasis on the step 2 CK exam. A study performed by Aziz et al. in 2021 determined the attitudes of general surgery program directors in regards to USMLE step 1 changing to pass/fail scoring. They concluded that not only will step 2CK scores become more emphasized, but that the medical school name, personal contacts, and clinical performance will hold more significance as well [25]. Program directors in the fields of otolaryngology, neurological surgery, plastic surgery, and orthopedic surgery also disagreed with the policy change based on studies from their respective specialty groups. Those in otolaryngology and orthopedic surgery agree that step 2CK and away rotations will have increasing importance [26,27]. It was determined that research output would become more emphasized in recruitment to neurological surgery programs [28]. Program directors in plastic surgery concluded that applicant familiarity as well as step 2CK scores are the only universal quantitative metrics that will form the main selection criteria [29]. These findings may lead to further disparities in osteopathic medical students' matching into surgical residency programs. In lieu of this change and the results of the current study, it only further suggests that osteopathic medical students should complete the USMLE exams and complete rotations to improve their chances of interviewing and matching at competitive surgical residency programs. Anecdotally, differing policies in regards to elective rotations, further exacerbated by the COVID-19 pandemic, may increase disparities for applicants regardless of the allopathic or osteopathic status of the medical school. More stringent policies may disallow opportunities for students to complete these rotations that many programs are stating as important.

While some institutions do not currently have an osteopathic resident within their program, it was indicated that they had simply not had many osteopathic candidates. This may be due to a lack of osteopathic medical schools in the area, exposure to certain programs, or an applicants' held bias that a lack of an osteopathic resident is a red flag. Based on the feedback received in this questionnaire, we suggest that osteopathic medical students focus on applying to programs that they are truly interested in, rather than assume programs will not give their application a fair review. As of 2021, there are currently thirty-seven osteopathic medical schools in fifty-eight teaching locations in thirty-three states [30]. Now that greater than 25% of all entering medical students are matriculating to osteopathic medical schools with increasing numbers of osteopathic graduates each year, some residents will have to be the first at their respective institutions [1].

Table 3 provides suggestions based on the literature review and results of the current study that osteopathic medical students and graduates should follow to improve their competitiveness for surgical residency programs. These are not inclusive of all opportunities that medical students may participate in, but provide a strong framework for students to follow.

**Table 3:** Overall suggestions for osteopathic medical students to increase their competitiveness to surgical residency programs. USMLE: United States Medical Licensing Exam; COMLEX: Comprehensive Osteopathic Medical Licensing Exam; NIH: National Institutes of Health; AMA: American Medical Association; ACOS: American College of Osteopathic Surgeons; ACS: American College of Surgeons.

Suggestion	Rationale	Examples
Osteopathic students should take USMLE step 1 and step 2 CK.	Many residency programs suggest or require these exams for adequate comparison of applicants despite recommendations that the COMLEX and USMLE be considered equal.	
Osteopathic students should seek out opportunities in basic science and clinical research.	Many programs and specialties have cited research as important or will have increased importance after transition of USMLE to a pass/fail scoring system.	Local institution, National Organizations (NIH, AMA), formal clinical research years at outside institutions, opportunities to share work through ACOS partnership with Cureus.
Osteopathic students should show an early interest in the specialty and programs of their choice.	Familiarity with an applicant will likely have an increased impact on applicant selection for interviews with fewer quantitative metrics to compare.	Social media interaction, program specific meet and greets/grand rounds, local, regional and national conference meetings, Apply for elective rotations and research opportunities.
Osteopathic students should foster mentorship relationships with senior students, residents, fellows and attendings from an early time in medical school.	These relationships can help in obtaining research, leadership positions, and overall development of a strong application to surgical residency programs.	National organization driven mentorship (ACOS, ACS), specialty specific driven mentorship, peer to peer medical student mentorship, outreach via social media, mentorship through online webinars provided by resident programs and specialty groups.
Osteopathic medical schools should develop affiliations with surgical residency programs and/or start their own program to create a pathway for osteopathic graduates.	There will always be an excess of applicants to surgical residency positions. With formation of new programs, further pathways can be created for medical graduates.	

Limitations

Given the nature in which the data were recorded by a questionnaire, the results may not be completely applicable to the residency programs that did not respond or provide feedback for osteopathic medical students to increase the competitiveness of their applications. There are several potential limitations to consider that may have led to a suboptimal response. These possibilities include inaccurate contact information on the ERAS profile, inability to reach a program contact due to an institutional firewall, or declining to respond. Additionally, only 28.3% of all responses were received from program directors of their residency programs. Though program coordinators are likely knowledgeable on program policies and recruitment strategies, there may still be different perceptions on what makes an applicant competitive between program directors and coordinators.

## Conclusions

For those who intend to apply to a surgical specialty, this study produced several actionable items to improve the competitiveness of their application, including completion of and performing well on USMLE step exams, completion of a sub-internship in the desired specialty and/or at a specific institution, and an increase in research experience and publications. Osteopathic medical students applying to surgical specialties should utilize all of the resources at their disposal to best prepare for application to competitive surgical residency programs.
